# Spatially resolved estimation of metabolic oxygen consumption from optical measurements in cortex

**DOI:** 10.1117/1.NPh.7.3.035005

**Published:** 2020-08-21

**Authors:** Marte J. Sætra, Andreas V. Solbrå, Anna Devor, Sava Sakadžić, Anders M. Dale, Gaute T. Einevoll

**Affiliations:** a University of Oslo, Centre for Integrative Neuroplasticity, Oslo, Norway; b University of Oslo, Department of Physics, Oslo, Norway; c Boston University, Department of Biomedical Engineering, Boston, Massachusets, United States; d Massachusetts General Hospital, Harvard Medical School, Athinoula A. Martinos Center for Biomedical Imaging, Charlestown, Massachusetts, United States; e University of California San Diego, Department of Radiology, La Jolla, California, United States; f University of California San Diego, Department of Neurosciences, La Jolla, California, United States; g Norwegian University of Life Sciences, Faculty of Science and Technology, Ås, Norway

**Keywords:** two-photon, oxygen, metabolism, analysis, estimation

## Abstract

**Significance:** The cerebral metabolic rate of oxygen (${\mathrm{CMRO}}_{2}$) is an important indicator of brain function and pathology. Knowledge about its magnitude is also required for proper interpretation of the blood oxygenation level-dependent (BOLD) signal measured with functional MRI. Despite the need for estimating ${\mathrm{CMRO}}_{2}$, no gold standard exists. Traditionally, the estimation of ${\mathrm{CMRO}}_{2}$ has been pursued with somewhat indirect approaches combining several different types of measurements with mathematical modeling of the underlying physiological processes. The recent ability to measure the level of oxygen (${\mathrm{pO}}_{2}$) in cortex with two-photon resolution in *in vivo* conditions has provided a more direct way for estimating ${\mathrm{CMRO}}_{2}$, but has so far only been used to estimate the average ${\mathrm{CMRO}}_{2}$ close to cortical penetrating arterioles in rats.

**Aim:** The aim of this study was to propose a method to provide spatial maps of ${\mathrm{CMRO}}_{2}$ based on two-photon ${\mathrm{pO}}_{2}$ measurements.

**Approach:** The method has two key steps. First, the ${\mathrm{pO}}_{2}$ maps are spatially smoothed to reduce the effects of noise in the measurements. Next, the Laplace operator (a double spatial derivative) in two spatial dimensions is applied on the smoothed ${\mathrm{pO}}_{2}$ maps to obtain spatially resolved ${\mathrm{CMRO}}_{2}$ estimates.

**Result:** The smoothing introduces a bias, and a balance must be found where the effects of the noise are sufficiently reduced without introducing too much bias. In this model-based study, we explored this balance using synthetic model-based data, that is, data where the spatial maps of ${\mathrm{CMRO}}_{2}$ were preset and thus known. The corresponding ${\mathrm{pO}}_{2}$ maps were found by solving the Poisson equation, which relates ${\mathrm{CMRO}}_{2}$ and ${\mathrm{pO}}_{2}$. MATLAB code for using the method is provided.

**Conclusion:** Through this model-based study, we propose a method for estimating ${\mathrm{CMRO}}_{2}$ with high spatial resolution based on measurements of ${\mathrm{pO}}_{2}$ in cerebral cortex.

## 1 Introduction

The level of consumption of oxygen by metabolic processes, that is, the cerebral metabolic rate of oxygen (${\mathrm{CMRO}}_{2}$), is an important indicator of brain function and pathology. Further, knowledge about the magnitude of the ${\mathrm{CMRO}}_{2}$ is required for a proper interpretation of the blood oxygenation level-dependent (BOLD) signal measured in functional MRI studies.^1^ The ability to measure ${\mathrm{CMRO}}_{2}$ with high spatial and temporal resolution in cortex is thus crucial. Traditionally, the ${\mathrm{CMRO}}_{2}$ has been estimated from several different types of measurements combined with mathematical modeling of the underlying physiological processes.^1^ Given the numerous assumptions and experimental limitations typically involved, questions have been raised about the accuracy of the estimates of the ${\mathrm{CMRO}}_{2}$ provided by these complex and somewhat indirect approaches.^2^

The possibility to optically measure the partial pressure of oxygen (${\mathrm{pO}}_{2}$) around cortical diving arterioles with two-photon resolution *in vivo*^3^ has provided a more direct way to estimate the ${\mathrm{CMRO}}_{2}$. Previously, we (Sakadžić, Devor, and collaborators) used measured ${\mathrm{pO}}_{2}$ gradients around diving arterioles in rats to estimate the average ${\mathrm{CMRO}}_{2}$ in the vessel’s vicinity, that is, within a radius of ∼100  μm.^4^ We based our estimates on the Krogh–Erlang formula relating the ${\mathrm{pO}}_{2}$ to the ${\mathrm{CMRO}}_{2}$ in a cylinder section around an arteriole providing the brain tissue with oxygen.^5^^,^^6^

The Krogh–Erlang formula assumes the ${\mathrm{pO}}_{2}$ level to have reached a stationary state, so that the fundamental equation relating the ${\mathrm{pO}}_{2}$ and the ${\mathrm{CMRO}}_{2}$ in the neural tissue can be described by the Poisson equation:

$$\nabla^{2}P(r)=M(r),$$ (1)

where P(r) represents ${\mathrm{pO}}_{2}$ measured at the position r, and M(r) is a measure that encapsulates the local ${\mathrm{CMRO}}_{2}$. The Krogh–Erlang formula gives a specific solution to the forward problem of this partial differential equation, that is, the radial profile of P, for the case where (i) the ${\mathrm{CMRO}}_{2}$ [M(r)] is assumed to be a constant and (ii) all the oxygen provided by the center arteriole is assumed to be consumed within a radial distance $R_{t}$.

The problem of estimating M(r) based on measured ${\mathrm{pO}}_{2}$ profiles P(r) is referred to as the inverse problem. In Ref. 4, this inverse problem was solved by fitting the Krogh–Erlang formula to ${\mathrm{pO}}_{2}$ data obtained in the close vicinity of a penetrating cerebral arteriole. This approach is global in the sense that it uses all measurements within a radial distance $R_{t}$ to obtain an estimate for an assumed constant value of M.

In this paper, we present a different approach to estimating M based on the same kind of two-photon ${\mathrm{pO}}_{2}$ measurements. The solution of inverse source problems for systems described by differential equations is important in many fields of science and technology and has consequently received substantial attention from mathematicians.^7^ Equation (1) is known as the Poisson equation, and several approaches have been taken to solve the inverse Poisson problem in different science and engineering contexts.^8^^–^^11^ In this study, we develop an approach to the inverse Poisson problem in the context of ${\mathrm{CMRO}}_{2}$ estimation. Specifically, we solve the problem by applying the Laplace operator $\nabla^{2}$ directly to suitably smoothed pressure maps P(r) to obtain a measure of M(r). We will refer to this approach as the diffusion-operator method for ${\mathrm{CMRO}}_{2}$ estimation. Unlike the Krogh–Erlang method, the diffusion-operator method provides a spatially resolved map of ${\mathrm{CMRO}}_{2}$ estimates around the arterioles and is thus not restricted to estimating an assumed constant value of M. Further, the diffusion-operator method is not restricted to situations with radially symmetric ${\mathrm{pO}}_{2}$ maps as when a single arteriole provides all oxygen.

The double spatial derivatives in the Laplace operator make the diffusion-operator method inherently very sensitive to noise in the measured spatial ${\mathrm{pO}}_{2}$ maps. In order to have a practical method for ${\mathrm{CMRO}}_{2}$ estimation, we smooth the experimental data in two dimensions before application of the Laplace operator to reduce the effects of the noise. Smoothing introduces a bias, that is, a systematic error in the estimates, and a balance must be found where the effects of the noise are sufficiently reduced without introducing too much bias. In the present model-based study we explore this balance by examining the accuracy of ${\mathrm{CMRO}}_{2}$ estimates in situations where the ground truth, that is, spatial maps of M(r) are preset and thus known, and the corresponding maps of P(r) are found by solving the forward problem of Eq. (1), either numerically or by taking advantage of the Krogh–Erlang formula.

The manuscript is organized as follows. In Sec. 2, we describe the diffusion-operator method, the methods used to provide model-based ${\mathrm{pO}}_{2}$ maps used in the method validation, and the metrics used to quantify the accuracy of the resulting estimates. In Sec. 3, we first illustrate the method and the necessary compromise between reducing noise and limiting bias when choosing the level of spatial smoothing. Next, we systematically explore the accuracy of ${\mathrm{CMRO}}_{2}$ estimates for a variety of situations with different levels of noise, different grid sizes of the ${\mathrm{pO}}_{2}$ measurement, and different levels of smoothing. In these systematic explorations of the efficacy of the method, the simple single-arteriole situation where the Krogh–Erlang formula gives the ground truth, is considered for simplicity. Later, we illustrate the use of the diffusion-operator method in more complicated situations where several arterioles provide the consumed oxygen, or the ${\mathrm{CMRO}}_{2}$ varies with position. In Sec. 4, we discuss the diffusion-operator method and its further development and use.

## 2 Methods

### 2.1 Mathematical Modeling of CMRO_2_ and pO_2_

The blood-tissue $O_{2}$ transport is thought to be dominated by diffusion.^12^ The relationship between ${\mathrm{pO}}_{2}$ values denoted as P(r,t) and the net rate of oxygen consumption s(r,t) in the tissue can then, in the general case, be described by^6^^,^^12^

$$\frac{\partial P(r,t)}{\partial t}=D\nabla^{2}P(r,t)+\frac{s(r,t)}{\alpha }.$$ (2)

In Eq. (2), $\nabla^{2}$ is the Laplace operator in three spatial dimensions (3D). Further, D and α are the diffusion coefficient and solubility, respectively, of oxygen in the tissue. They are assumed to be space-invariant. If warranted, Eq. (2) can be generalized to the case where D depends on position and direction, or when α varies with position.^12^

Equation (2) is only applicable outside the arterioles supplying the oxygen to the brain tissue. In the context of this equation, the oxygen supplied to the tissue is represented by a boundary condition of ${\mathrm{pO}}_{2}$ imposed at the vessel wall of the arteriole. Note, however, that the effect of an oxygen supply from a bed of small capillary vessels located some distance away from the arteriole may be incorporated in the description. Such an oxygen supply will offset (or even reverse the sign of) the net rate of oxygen consumption s(r,t) in this region.

In this paper, we will focus on a special case of this diffusion problem where (i) the system is in a steady-state so that the term ∂P(r,t)/∂t can be neglected and (ii) there is no variation of ${\mathrm{pO}}_{2}$ in the vertical z-direction, that is, the direction along the cortical axis parallel to the penetrating arteriole. These assumptions are also incorporated in the Krogh–Erlang model used to estimate the ${\mathrm{CMRO}}_{2}$ in Ref. 4. In this case, the diffusion equation [Eq. (2)] simplifies to

$$\nabla^{2}P(r)=\frac{s(r)}{D\alpha },$$ (3)

where $\nabla^{2}$ now refers to the 2D Laplace operator (which with Cartesian coordinates reads $\nabla^{2}=\partial^{2}/\partial x^{2}+\partial^{2}/\partial y^{2}$). Equation 3 can be written more compactly as

$$\nabla^{2}P(r)=M(r),$$ (4)

where

M(r)≡s(r)/Dα. (5)

Here M(r) is a new position-dependent variable encapsulating the net rate of oxygen consumption in the neural tissue. In principle, Eq. (4) describes the spatial map of ${\mathrm{pO}}_{2}$ for any set of oxygen sinks [metabolic consumption, s(r)>0] and sources [i.e., oxygen provided by small capillaries, s(r)<0]. The variable M(r) is then proportional to the net rate of oxygen consumption, that is, the difference between oxygen sinks and sources at position r in tissue.

By introducing a characteristic length $r^{*}$ and a characteristic oxygen consumption $M^{*}$, we can rewrite Eq. (4) in a dimensionless form which is useful in the further analysis:

∇^2P^(r^)=M^(r^). (6)

In Eq. (6), r^=r/r*, $\hat{P}=P/(M^{*}r^{*2})$, $\hat{M}=M/M^{*}$, and ∇^2 is the Laplace operator in terms of the dimensionless position variables. In this dimensionless form, the number of model parameters is effectively reduced by one, making the further analysis simpler.

### 2.2 Inverse Problem of Estimating CMRO_2_ from pO_2_ Measurements

We estimate ${\mathrm{CMRO}}_{2}$ by solving the inverse diffusion problem, that is, the problem where P(r) is known from experiments, and the net rate of oxygen consumption s(r,t) is the unknown function of interest. It follows from Eq. (2) that s(r,t) based on ${\mathrm{pO}}_{2}$ measurements $P_{\text{data}}(r,t)$ is given by

$$s_{\mathrm{est}}(r,t)=\alpha \frac{\partial P_{\text{data}}(r,t)}{\partial t}+\alpha D\nabla^{2}P_{\text{data}}(r,t).$$ (7)

For the stationary 2D case, this reduces to

$$s_{\mathrm{est}}(r)=\alpha D\nabla^{2}P_{\text{data}}(r),$$ (8)

or

$$M_{\mathrm{est}}(r)=\nabla^{2}P_{\text{data}}(r),$$ (9)

where $\nabla^{2}$ is the Laplace operator in 2D.

Equation 9 says that given a data set of oxygen partial pressure $P_{\text{data}}$ measured on a 2D spatial grid, M can be estimated by taking the Laplacian of $P_{\text{data}}$ (or in practice, a smoothed version of $P_{\text{data}}$). We refer to this approach as the diffusion-operator method for ${\mathrm{CMRO}}_{2}$ estimation. If one wants estimates for $s_{\mathrm{est}}$, values of the diffusion coefficient D and the solubility α are also required.

The double spatial derivatives in the Laplace operator make the diffusion-operator method inherently very sensitive to noise in the measured spatial ${\mathrm{pO}}_{2}$ profiles. Thus to reduce adverse effects of noise in the ${\mathrm{pO}}_{2}$ measurements, we pursue a method that spatially smooths $P_{\text{data}}$ before application of the Laplace operator.

#### 2.2.1 Smoothing of pO_2_ data

To smooth pressure data, we performed cubic smoothing spline interpolation using the csaps function in MATLAB’s Curve Fitting Toolbox. The function minimizes the square deviation between the estimated and measured 2D data (so-called L2 norm) while penalizing large double-spatial derivatives in the smoothed data. Other smoothing procedures could have been pursued instead, but a key motivation for this particular choice was the public availability of the tool.

In terms of dimensionless quantities, the csaps function takes a given data set P^data(x^,y^) and generates a smoothing spline P^smooth(x^,y^) that minimizes

(1−q)∑i=1n∑j=1m[P^data(x^i,y^j)−P^smooth(x^i,y^j)]2+q∬{[∂2P^smooth(x^,y^)∂x^2]2+[∂2P^smooth(x^,y^)∂y^2]2}dx^ dy^. (10)

Here n and m are the number of entries of $\hat{x}$ and $\hat{y}$, respectively, and q is a smoothing parameter between 0 and 1. q=0 corresponds to the case with no smoothing, and increasing values of q imply increasing the amount of smoothing. Note that the csaps function takes p=1−q as input argument, see MATLAB documentation. This MATLAB function allows for giving more weights to some data points than others in the optimization. We keep the weights identical to 1 for all data points in the present application.

The csaps function allows the smoothing spline P^smooth to be computed with higher resolution than the spatial resolution of the measurements. This is convenient as it allows for a higher spatial resolution in the maps of estimated M obtained from the discrete Laplace function del2. Assuming that the measurements are taken in a rectangular grid of points,^13^ we here refer to the grid spacing between the pressure data points as d^data, and the grid spacing of the estimated pressure points P^smooth as ${\hat{d}}_{\mathrm{est}}$. In the smoothing function, ${\hat{d}}_{\mathrm{est}}$ is set by inserting position vectors for the estimation points ${\hat{x}}_{\mathrm{est}}$ and ${\hat{y}}_{\mathrm{est}}$ with this spacing. Likewise, d^data is set by inserting position vectors for the data points $\hat{x}$ and $\hat{y}$ with this spacing. Then P^smooth is estimated from the recorded pressure by the following call of csaps:

P^smooth=csaps({y^,x^},P^,(1−q),{y^est,x^est}). (11)

In this paper, we keep a fixed small value of ${\hat{d}}_{\mathrm{est}}$, that is, ${\hat{d}}_{\mathrm{est}}=0.001$, to minimize the error introduced from the discreteness of the Laplace operator used in the estimator presented in the next section. With this choice, the discreteness error is negligible far away from the arteriole and much smaller than other estimation errors close to the arteriole.

#### 2.2.2 Application of Laplace operator

After the smoothing procedure, the net oxygen consumption as described by $\hat{M}(\hat{x},\hat{y})$ can be estimated directly by application of the Laplace operator:

M^est(x^,y^)=∇^2P^smooth(x^,y^). (12)

With P^smooth given on a square (or rectangular) grid with grid spacing $\hat{d}$, we apply the discrete finite difference approximation of the Laplace operator:

M^est(x^i,y^j)=P^smooth(x^i+1,y^j)+P^smooth(x^i−1,y^j)+P^smooth(x^i,y^j+1)+P^smooth(x^i,y^j−1)−4P^smooth(x^i,y^j)d^2. (13)

Here the integers i and j represent the grid point positions, that is, ${\hat{x}}_{i}=i\hat{d}$ and ${\hat{y}}_{j}=j\hat{d}$. In the present application, the MATLAB function del2 is used to compute this discrete finite difference approximation of the Laplace operator. Note that in order to calculate the right-hand side of Eq. (13), one must multiply the output from del2 by 4. Specifically, we use the command 4*del2(P^smooth,d^) to calculate ${\hat{M}}_{\mathrm{est}}(\hat{x},\hat{y})$.

#### 2.2.3 Choice of smoothing parameter

The effect of the csaps smoothing function can be characterized by a smoothing length ${\hat{d}}_{q}$, which describes how much a spatial δ-function is smeared out in space. By numerical exploration, we found that this characteristic smoothing length depends on q and d^data through the relationship

d^q=k(qd^data)1/4, (14)

where k is a constant.

**Fig. 1:**
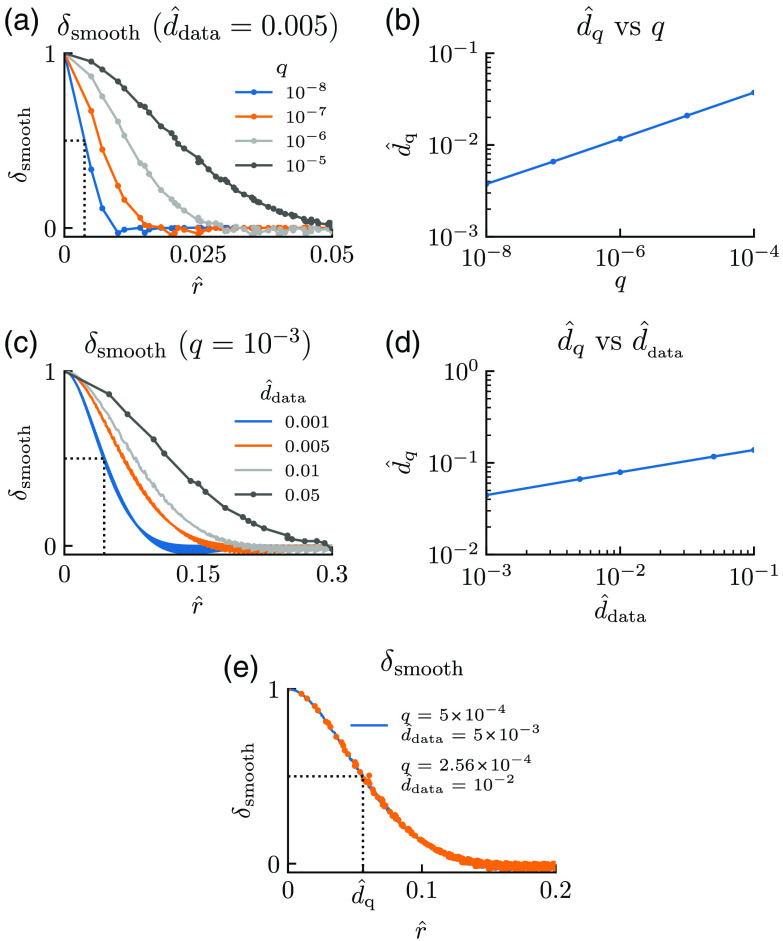
Choice of smoothing parameter in csaps. The effect of the smoothing function csaps is characterized by a smoothing length ${\hat{d}}_{q}$ that is related to the smoothing factor q and the spatial spacing d^data through Eq. (14). We found this relationship by smoothing a 2D spatial δ-function using different values of q and d^data, and plotting the result as a function of the distance $\hat{r}$ from the position of the δ-function. (a) and (c) The normalized smoothed δ-function [δsmooth(r^)] for different values of q (d^data fixed) and d^data (q fixed), respectively. The characteristic smoothing length ${\hat{d}}_{q}$ is defined as the distance corresponding to $\delta_{\text{smooth}}=0.5$ (dotted lines) and is plotted as a function of q and d^data in (b) and (d), respectively. In (e), we demonstrate how different sets of q and d^data-values correspond to the same ${\hat{d}}_{q}$, that is, the same smoothing effect.

This relationship was found numerically by smoothing a square single-entry matrix with one as the center element, and the rest of the elements set to zero. The resulting spatially smoothed δ-function was then plotted, for a fixed value of d^data and different values of q, as a function of the distance r to the center point, as shown in Fig. 1(a). We then defined the characteristic length ${\hat{d}}_{q}$ to be the distance from the center point, in which the function value had fallen 50% compared to the center value, see dotted lines in Fig. 1(a). Figure 1(b) shows the dependence of the estimated ${\hat{d}}_{q}$ on q (for a fixed d^data of 0.005). We observe that ${\hat{d}}_{q}$ increases slowly with q, that is, when q is increased by a factor $10^{4}$, ${\hat{d}}_{q}$ increases only by a factor 10. Figure 1(c) shows the smoothed δ-function when instead the value of q is fixed, while d^data has different values. Again, when ${\hat{d}}_{q}$ is read out from the curve and plotted as a function of d^data [Fig. 1(d)], we see that ${\hat{d}}_{q}$ increases slowly with d^data, that is, when d^data is increased by a factor $10^{4}$, ${\hat{d}}_{q}$ increases only by a factor 10.

The detailed value of the constant k in Eq. (14) is not critical for our purpose. We set it by reading out the value for ${\hat{d}}_{q}$ from the graph for the case with d^data=5×10−3 and $q=5\times 10^{-4}$ as shown with a blue line in Fig. 1(e). The readout value, d^q≈5.6×10−2, was then used to calculate k from Eq. (14). After rounding to one decimal, this gave k=1.4.

Thus given d^data and a chosen value of ${\hat{d}}_{q}$, we can find which q to use in csaps in Eq. (11) through the following equation:

q=(d^q1.4)41d^data. (15)

This equation tells us that if, say, d^data increases from $5\times 10^{-3}$ to $1\times 10^{-2}$, then q must decrease from $q=5\times 10^{-4}$ to about $q=2.6\times 10^{-4}$ to keep the same smoothing effect, that is, give the same value of ${\hat{d}}_{q}$. The dotted orange line in Fig. 1(e) illustrates that this is indeed the case.

### 2.3 Forward Modeling of Ground Truth pO_2_ Data

To validate the ${\mathrm{CMRO}}_{2}$ estimation method, we generate synthetic data of oxygen partial pressure P^(r^) by solving Eq. (4) for chosen values of M^(r^) and chosen geometries of vascular sources and measurements points. The synthetic data work as a “ground truth.” Since we know its true value of M^(r^), we can use it to test our estimation method. In this study, we compute this ground truth data by means of two methods: (i) using the Krogh–Erlang model and (ii) by means of finite-element modeling.

#### 2.3.1 Krogh–Erlang model

In the well-known Krogh–Erlang model,^5^ a cylindrical geometry, mimicking a straight segment of a blood vessel, was used to model the metabolic consumption of oxygen provided by capillaries in muscles. In Ref. 4, the same model was used to study metabolic consumption of oxygen provided by penetrating arterioles in brain tissue. The model describes the blood vessel as a small cylinder with radius $R_{\mathrm{ves}}$ supplying a tissue cylinder with radius $R_{t}$ with oxygen. The further assumptions are (i) uniform consumption of oxygen in the tissue, that is, constant M outside the vessel, (ii) no axial diffusion of oxygen, (iii) $P=P_{\mathrm{ves}}$ at $R_{\mathrm{ves}}$, and (iv) no pressure gradient at the surface of the tissue cylinder, that is, dP/dr=0 at $R_{t}$. With these four assumptions, the solution of Eq. (4) is found to be

$$P(r)=P_{\mathrm{ves}}+\frac{1}{4}M(r^{2}-R_{\mathrm{ves}}^{2})-\frac{1}{2}MR_{t}^{2}\;\ln\;\frac{r}{R_{\mathrm{ves}}},$$ (16)

for $R_{t}\geq r\geq R_{\mathrm{ves}}$. This so-called Krogh–Erlang formula predicts the oxygen pressure P in the tissue as a function of the distance r from the vessel’s center. For our application, we set $P(r)=P_{\mathrm{ves}}$ if $r<R_{\mathrm{ves}}$.

Equation 16 can be written in dimensionless form as

P^(r^)={P^ves,if  r<R^vesP^ves+14M^(r^2−R^ves2)−12M^R^t2 ln r^R^ves,if  R^t≥r≥R^ves. (17)

Here we also have introduced ${\hat{P}}_{\mathrm{ves}}=P_{\mathrm{ves}}/(M^{*}r^{*2})$, $\hat{r}=r/r^{*}$, ${\hat{R}}_{\mathrm{ves}}=R_{\mathrm{ves}}/r^{*}$, and ${\hat{R}}_{t}=R_{t}/r^{*}$. Further, the boundary condition $d\hat{P}/d\hat{r}=0$ for $\hat{r}={\hat{R}}_{t}$ is assumed.

#### 2.3.2 Finite-element modeling: FEniCS model

The Krogh–Erlang formula relates the oxygen consumption and the partial oxygen pressure under very specific conditions. Another option is to solve Eq. (6) numerically. This allows for the solutions for more general cases, such as a more complicated geometry with, for example, several arterioles providing oxygen, or an inhomogenous oxygen consumption. We implemented Eq. (6) in the finite-element software package FEniCS^14^ and verified the implementation by comparing the result to that of the Krogh–Erlang formula.

The FEniCS implementation solves the variational formulation of Eq. (6): Let V be a space of test functions $\left\{v_{1},\ldots v_{N}\right\}$ on the computational domain Ω. We aim to find $\hat{P}$ such that

∫Ω∇P^·∇vi+M^vidx−∫∂Ω∇P^·nds=0,∀  vi∈V, (18)

where ∂Ω denotes the boundary of the domain, and n is a normal vector pointing out of the domain. This variational form is obtained by multiplying Eq. (6) with the test function $v_{i}$ and integrating over Ω, followed by integration by parts of the Laplacian term. Note that as we apply a fixed value for $\hat{P}$ by the blood vessel and no pressure gradient at the boundary of the domain, the boundary integral in Eq. (18) vanishes.

The solution to Eq. (18) gives us $\hat{P}$ on an unstructured finite-element mesh. Experimental data are typically measured on a structured Cartesian grid, and to better mimic this we transfer the synthetic data generated by FEniCS to a 2D NumPy array. We do this by first defining a new Cartesian mesh using NumPy with a distance d^data between each point. Then in the next step, we pick out values of $\hat{P}$ from the FEniCS solution corresponding to these positions and save them to a 2D NumPy array. We set $P(r)=P_{\mathrm{ves}}$ if $r<R_{\mathrm{ves}}$.

#### 2.3.3 Noise

We add additive Gaussian noise to the synthetic data using the normrnd function in MATLAB. For each value $\hat{P}$ of oxygen partial pressure, whether it comes from the Krogh–Erlang equation or the FEniCS solution, we draw a random number from a Gaussian distribution with mean $\hat{P}$ and standard deviation (SD) ${\hat{\sigma }}_{P}$, and replace $\hat{P}$ by this number.

### 2.4 Performance Measures of the Diffusion-Operator Method

In order to evaluate the performance of the diffusion-operator method, we test it on the synthetic data and calculate its bias, precision, and accuracy. As precision and accuracy measures, we use SD and root-mean-square error (RMSE). The mathematical definitions of these measures are

bias=1N∑j=1N(M^est,j−M^), (19)

SD=1N∑j=1N(M^est,j−M^est¯)2, (20)

and

RMSE=1N∑j=1N(M^est,j−M^)2, (21)

where N is the number of synthetic samples and ${\hat{M}}_{\mathrm{est},j}$ is the j’th estimate of $\hat{M}$.

The RMSE combines both bias and precision as its squared value MSE is equal to the SD squared plus the bias squared: $\mathrm{MSE}={\mathrm{SD}}^{2}+{\text{bias}}^{2}$.^15^

## 3 Results

### 3.1 Illustration of the Diffusion-Operator Method

**Fig. 2:**
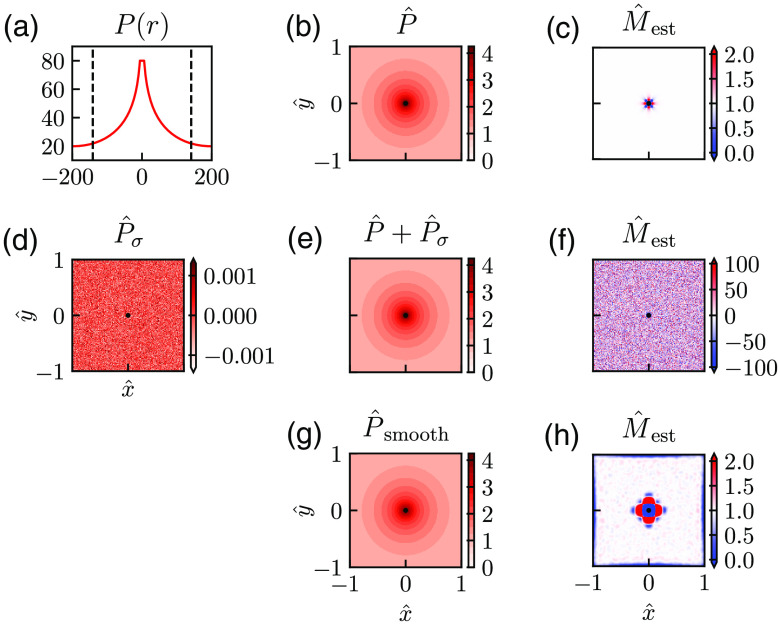
Illustration of diffusion-operator estimation method. (a) An example of a synthetic ${\mathrm{pO}}_{2}$ profile calculated using the Krogh–Erlang formula in Eq. (17) without noise. (b) The corresponding 2D representation of this ${\mathrm{pO}}_{2}$ data set with use of dimensionless parameters. (d) A map of additive Gaussian noise ${\hat{P}}_{\sigma }$ and (e) the corresponding ${\mathrm{pO}}_{2}$ map where this noise has been added. (g) A data set where smoothing has been applied. (c), (f), and (h) Estimated M^s calculated from the ${\mathrm{pO}}_{2}$ data in (b), (e), and (g), respectively. Parameter values: all panels: $P_{\mathrm{ves}}=80\;\mathrm{mmHg}$, $R_{\mathrm{ves}}=6\;\mu m$, $R_{t}=200\;\mu m$, $M=0.001\;\mathrm{mm}\mathrm{Hg}\;\mu m^{-2}$. (a) $d_{\text{data}}=1\;\mu m$; (b)–(h) d^data=0.007; $r^{*}=141\;\mu m$, $M^{*}=M$; (d)–(h) σ^P=5×10−4; and (g), (h) ${\hat{d}}_{\mathrm{est}}=0.001$, ${\hat{d}}_{q}=0.04$.

The principle of the diffusion-operator method for estimation of the net oxygen consumption M(r) from ${\mathrm{pO}}_{2}$ measurements P(r) is illustrated in Fig. 2. In this example, we assume the spatial map of ${\mathrm{pO}}_{2}$ to follow the Krogh–Erlang formula in Eq. (16), mimicking the situation where a single arteriole is the source of the oxygen, and the oxygen consumption M is constant around the arteriole.

Figure 2(a) shows the pressure profile in the radial directions as described by this formula with example parameters $P_{\mathrm{ves}}$, M, and $R_{\mathrm{ves}}$ chosen to be in qualitative agreement with example data from Ref. 4, that is, $P_{\mathrm{ves}}=80\;\mathrm{mmHg}$, $M=0.001\;\mathrm{mmHg}\;\mu m^{-2}$, and $R_{\mathrm{ves}}=6\;\mu m$, and $R_{t}$ set to 200  μm. Figure 2(b) shows a contour plot of this synthetic ${\mathrm{pO}}_{2}$ map in 2D. Here dimensionless parameters (cf., Sec. 2) are used with $r^{*}=141\;\mu m$ and the convenient choice $M^{*}=M$ so that the maximal pressure $P_{\mathrm{ves}}$ corresponds to ${\hat{P}}_{\mathrm{ves}}\approx 4.1$ and $\hat{M}=1$. We show the ${\mathrm{pO}}_{2}$ map in a square window with side lengths of 282  μm so that the dimensionless position coordinates extends from −1 to 1 along the $\hat{x}$ and $\hat{y}$ axes. With this choice, the corners of the square correspond to a radial distance equal to ${\hat{R}}_{t}$, the radius of the tissue cylinder.

The problem of ${\mathrm{CMRO}}_{2}$ estimation now corresponds to estimating M at the different locations inside the square window based on these recordings. Figure 2(c) shows the estimated M (in units of $M^{*}$) found by applying the Laplace estimator in Eq. (13) on the data in Fig. 2(b). In this example, the dimensionless distance between the grid points, in which ${\mathrm{pO}}_{2}$ is “measured” is set to $\hat{d}=0.007$, corresponding to a physical grid-point distance of about 1  μm. It is seen that some distance away from the vessel, the estimator predicts $\hat{M}$ very close to 1, that is, $M≃M^{*}$, as it should.

However, close to the vessel, that is, for $\hat{r}≳{\hat{R}}_{\mathrm{ves}}$, clearly incorrect values of $\hat{M}$ are obtained. One obvious reason is that the discrete Laplace estimator in Eq. (13) will be inaccurate when one or more of the grid points used in the estimation is inside the vessel. Here the pressure P is not described by Eq. (16) and is instead assumed constant so that $\nabla^{2}P\neq M$, cf. Eq. (4). For the present example, a more important reason is that immediately outside the vessel, the pressure profile drops sharply [due to the last term in the Krogh–Erlang formula in Eq. (16)] so that the discrete Laplace estimator becomes inaccurate when the grid-point distance $\hat{d}$ is too large. The “flower-like” symmetric pattern of this estimation error in Fig. 2(c) reflects the Cartesian symmetry of the estimator in Eq. (13). This discretization error can be reduced by reducing the value of $\hat{d}$, i.e., using a finer grid.

Figure 2(c) illustrates that if the experimental measurements were noiseless, the Laplace estimator in Eq. (13) could be used directly on the ${\mathrm{pO}}_{2}$ data, at least when the grid of recordings is finely spaced. This would apply for any distribution of vessels as long as the estimator ${\hat{M}}_{\mathrm{est}}$ in Eq. (13) is used sufficiently far away from the vessel wall. Experimental ${\mathrm{pO}}_{2}$ data will always contain noise, however, and Fig. 2(d) shows a map of additive Gaussian noise ${\hat{P}}_{\sigma }$ with zero mean and SD ${\hat{\sigma }}_{P}=0.0005$. Figure 2(e) shows the same synthetic data as in Fig. 2(b) where this noise has been added, indistinguishable by eye from the noise-free map in Fig. 2(b). When ${\hat{M}}_{\mathrm{est}}$ in Eq. (13) is applied on these synthetic data, the estimated values of $\hat{M}$ are wildly inaccurate [Fig. 2(f)]. Not only does the estimated values of $\hat{M}$ have much larger magnitudes than the true value of $\hat{M}=1$, they also have both signs and vary strongly between neighboring grid positions (that is, between neighboring pixels in the map). These poor estimates reflect that the double-derivative operation of the Laplacian estimator corresponds to a high-pass spatial filtering that effectively amplifies the effects of the noise in the data.

This noise in the estimated $\hat{M}$ can be reduced by the use of spatial smoothing, that is, low-pass filtering, of the data $\hat{P}$ before application of ${\hat{M}}_{\mathrm{est}}$. While the smoothed map P^smooth in Fig. 2(g) at first glance does not appear to be very different from the unsmoothed version in Fig. 2(e), the effect of the smoothing on the estimated M is dramatic [(Fig. 2(h)]. With the choice of smoothing used in this example (see figure caption for details), quite accurate estimates of $\hat{M}$ are found for a large region of the area around the central vessel [light-colored regions of Fig. 2(h)]. However, the smoothing procedure results in large estimation errors in a sizable region around the blood vessel as well as close to the edges of the square data set.

To summarize, suitable smoothing of the ${\mathrm{pO}}_{2}$ data before using the Laplace estimator ${\hat{M}}_{\mathrm{est}}$ may dramatically improve the estimation accuracy. However, the choice of smoothing is critical: too little low-pass smoothing will not remove enough of the high-frequency spatial noise; too much smoothing will smooth away spatial information in the data and thus give poor estimates of M. Next, we will investigate this dilemma in more detail.

### 3.2 Noise Removal versus Bias

**Fig. 3:**
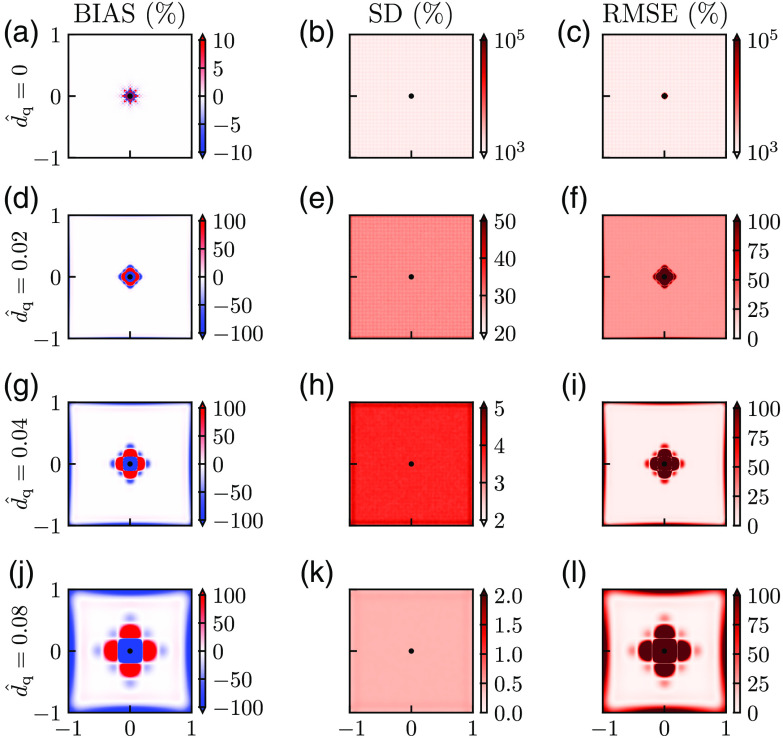
Illustration of noise removal versus bias. (a), (d), (g), and (j) Bias of $M_{\mathrm{est}}$ for different values of $d_{q}$. (b), (e), (h), and (k) SD of $M_{\mathrm{est}}$ for different values of $d_{q}$. (c), (f), (i), and (l) RMSE of $M_{\mathrm{est}}$ for different values of $d_{q}$. Bias is computed from Eq. (19) for the case without noise ${\hat{\sigma }}_{P}=0$ so that a single estimate of ${\hat{M}}_{\mathrm{est}}$ is sufficient, that is N=1 in Eq. (19). SD is computed from Eq. (20) with $10^{4}$ estimates of ${\hat{M}}_{\mathrm{est}}$, that is, $N=10^{4}$. In the computation of SD and RMSE, σ^P=5×10−4. All performance measures are given as the percentage of the ground truth value $\hat{M}=1$. Note also that the MATLAB routine csaps is used also for the case without smoothing (d^q=0) with q=0 inserted in Eq. (11). Other parameter values: d^data=0.007, $P_{\mathrm{ves}}=80\;\mathrm{mmHg}$, $R_{\mathrm{ves}}=6\;\mu m$, $R_{t}=200\;\mu m$, $M=0.001\;\mathrm{mmHg}\;\mu m^{-2}$, $r^{*}=141\;\mu m$, and $M^{*}=M$.

Figure 3 illustrates the dilemma when choosing the right level of low-pass smoothing of the ${\mathrm{pO}}_{2}$ data P before using the Laplace estimator in Eq. (13). In the smoothing algorithm, the quantity described in Eq. (10) was minimized to penalize sharp variations in $P_{\text{smooth}}$ while at the same time fitting the synthetic data $P_{\text{data}}$. The level of smoothing is set by the smoothing length $d_{q}$ (or ${\hat{d}}_{q}$ in dimensionless units) which is related to the smoothing parameter used in the presently used MATLAB function csaps via Eq. (14) (see Sec. 2). This smoothing length describes how much a point (that is, a 2D spatial δ-function) will be smeared out in space. Thus the larger $d_{q}$ is, the more the ${\mathrm{pO}}_{2}$ map will be smeared out or smoothed.

To quantify the performance of the estimator, we use the following three performance measures: bias, SD, and RMSE. The bias [Eq. (19)] measures the systematic error in the estimator $M_{\mathrm{est}}$ introduced by the smoothing (and discreteness of data points) whether the data is noisy or not. It can be evaluated from noiseless data (that is, with $P_{\sigma }=0$), and the results for different values of smoothing are shown in Figs. 3(a), (d), (g), and (j). In the case of no smoothing [${\hat{d}}_{q}=0$, Fig. 3(a)], the only bias comes from the discreteness of the grid of data points and is only observed close to the vessel. With a small amount of smoothing [${\hat{d}}_{q}=0.02$, Fig. 3(d)], the bias around the vessel is increased. For ${\hat{d}}_{q}=0.04$ [Fig. 3(g)] and d^q=0.08 [Fig. 3(j)], this tendency of increased bias with increasing ${\hat{d}}_{q}$ is continued, and some bias is also observed close to the edges of the square grid. For the largest smoothing depicted in Fig. 3(j), about one-third or so of the map has a bias with a magnitude larger than 100%.

The SD [Eq. (20)] measures the precision or the error in the estimation due to the presence of noise. This measure obviously depends on the level of noise $P_{\sigma }$. In the present example in Fig. 3, a Gaussian noise with a SD of ${\hat{\sigma }}_{P}=5\times 10^{-4}$ is used. With $r^{*}=141\;\mu m$ and $M^{*}=0.001\;\mathrm{mmHg}\;\mu m^{-2}$ as in Fig. 2 this corresponds to a physical noise level of $\sigma_{P}\approx 0.01\;\mathrm{mmHg}$. The SD for different amounts of smoothing is shown in Figs. 3(b), (e), (h), and (k). Three observations of note are that (i) the SD of the estimates is extremely large when no smoothing is applied (${\hat{d}}_{q}=0$), (ii) the SD decreases with increasing ${\hat{d}}_{q}$, and (iii) unlike for the bias, the SD has similar values at the different positions.

An essential feature of the SD is that it is proportional to the SD of the noise in the pressure ${\hat{\sigma }}_{P}$. Thus if ${\hat{\sigma }}_{P}$ was doubled to 0.001, the SDs in Figs. 3(b), (e), (h), and (k) would be doubled as well.

The accuracy of the estimator $M_{\mathrm{est}}$ is measured by the RMSE [Eq. (21)], which incorporates both the bias and precision (SD) through the relation

$$\mathrm{RMSE}=\sqrt{{\text{bias}}^{2}+{\mathrm{SD}}^{2}}.$$ (22)

This measure describes the total statistical uncertainty of the estimates when $M_{\mathrm{est}}$ is applied on individual data sets. The bias increases with increasing ${\hat{d}}_{q}$ [Figs. 3(a), (d), (g), and (j)], whereas the SD instead decreases with increasing ${\hat{d}}_{q}$ [Figs. 3(b), (e), (h), and (k)]. One would thus expect a suitable intermediate value of ${\hat{d}}_{q}$ to give the smallest RMSE. For the example in Fig. 3, we indeed see that for the values of ${\hat{d}}_{q}$ considered, the intermediate value ${\hat{d}}_{q}=0.04$ [Fig. 3(I)] offers the best compromise between bias and noise removal and gives the smallest RMSE. For this value of ${\hat{d}}_{q}$, the RMSE is smaller than 25% for almost all positions except for a region around the blood vessel.

The large RMSE close to the blood vessel even for the “best” choice of ${\hat{d}}_{q}$ in Fig. 3(I) reflects the large bias at these locations [Fig. 3(g)].

### 3.3 Choice of Smoothing Length $d_{q}$

As illustrated in the previous section, a key question when using the Laplace estimator in Eq. (13) is the choice of the amount of smoothing, or more specifically, the choice of the smoothing length $d_{q}$. This will not only depend on the noise level, but also the spatial resolution of the data as described by the grid resolution, that is, the distance between adjacent points on the measurement grid, $d_{\text{data}}$. Since the bias is independent of the noise level, and the SD is linearly proportional to the SD $\sigma_{P}$ of the noise, it is convenient to first explore the interplay between $d_{q}$ and $d_{\text{data}}$ for the bias and SD separately.

**Fig. 4:**
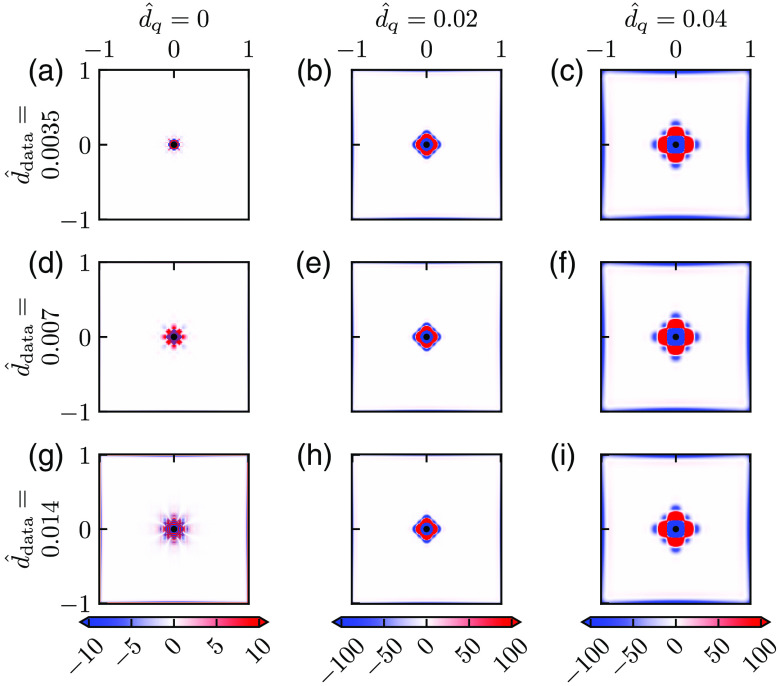
Bias for different smoothing. (a)–(i) Bias of $M_{\mathrm{est}}$ for different values of $d_{q}$ (increasing from left to right) and ${\hat{d}}_{\mathrm{est}}=0.001$ (increasing from top to bottom) computed from Eq. (19) and given as the percentage of the ground truth value $\hat{M}=1$. There was no noise added to the pressure data so that a single estimate of ${\hat{M}}_{\mathrm{est}}$ is sufficient, that is N=1 in Eq. (19). Parameter values: $P_{\mathrm{ves}}=80\;\mathrm{mmHg}$, $R_{\mathrm{ves}}=6\;\mu m$, $R_{t}=200\;\mu m$, $M=0.001\;\mathrm{mmHg}\;\mu m^{-2}$, $r^{*}=141\;\mu m$, and $M^{*}=M$.

In Fig. 4, we show how the bias varies with $d_{\text{data}}$ and $d_{q}$ for three choices of parameter values of each: d^data=0.0035, 0.007, 0.014 (here corresponding to physical grid resolutions of approximately 0.5, 1, and 2  μm, respectively), ${\hat{d}}_{q}=0$, 0.02, and 0.04 (corresponding to physical smoothing lengths of approximately 0, 3, and 6  μm, respectively). For the case with no smoothing [Figs. 4(a), (d), and (g)], we observe that the bias increases with increasing d^data. This illustrates that the error due to the discreteness of the Laplace estimator is sensitive to $d_{\text{data}}$ even when $d_{\mathrm{est}}$ is set to a very small number (${\hat{d}}_{\mathrm{est}}=0.001$, cf., Sec. 2). This is not surprising because decreasing the grid resolution from d^data to ${\hat{d}}_{\mathrm{est}}$ means that we estimate $\hat{P}$ at a denser grid of points than what is directly available in the data. With smoothing added (two rightmost columns of Fig. 4), the bias increases, and the larger the value of ${\hat{d}}_{q}$ is, the larger the bias is. (Note the difference in color scales in this figure.)

**Fig. 5:**
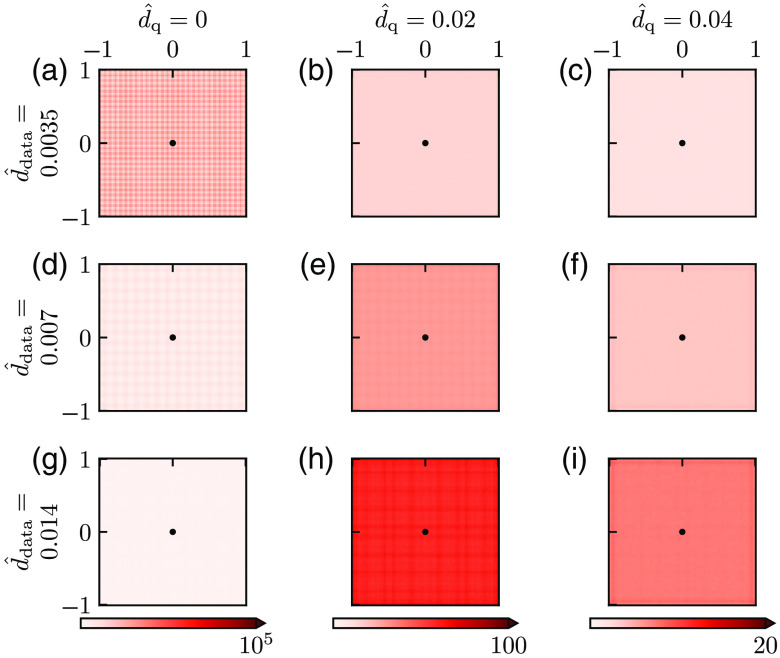
SD for different smoothing—fixed noise level. (a)–(i) SD of $M_{\mathrm{est}}$ for different values of $d_{q}$ (increasing from left to right) and $d_{\text{data}}$ (increasing from top to bottom) computed from Eq. (20) with $N=10^{4}$. Values are given as the percentage of the ground truth value $\hat{M}=1$. (Note that the grid-like pattern visible in some of the panels is a numerical artifact resulting from the application of the MATLAB routine csaps.) Parameter values: σ^P=5×10−4, $P_{\mathrm{ves}}=80\;\mathrm{mmHg}$, $R_{\mathrm{ves}}=6\;\mu m$, $R_{t}=200\;\mu m$, $M=0.001\;\mathrm{mmHg}\;\mu m^{-2}$, $r^{*}=141\;\mu m$, and $M^{*}=M$.

In Fig. 5, we correspondingly show how the SD varies with $d_{\text{data}}$ and $d_{q}$ for the same set of parameters as in Fig. 4 for a fixed level of noise in the data, ${\hat{\sigma }}_{P}=5\times 10^{-4}$. Here the most important feature is that the SD is strongly reduced with increased smoothing, that is, increasing $d_{q}$ (from left to right). For the smoothed cases (two rightmost columns), we also observe that SD increases with increasing $d_{\text{data}}$ (i.e., making the grid of measurements more sparse).

**Fig. 6:**
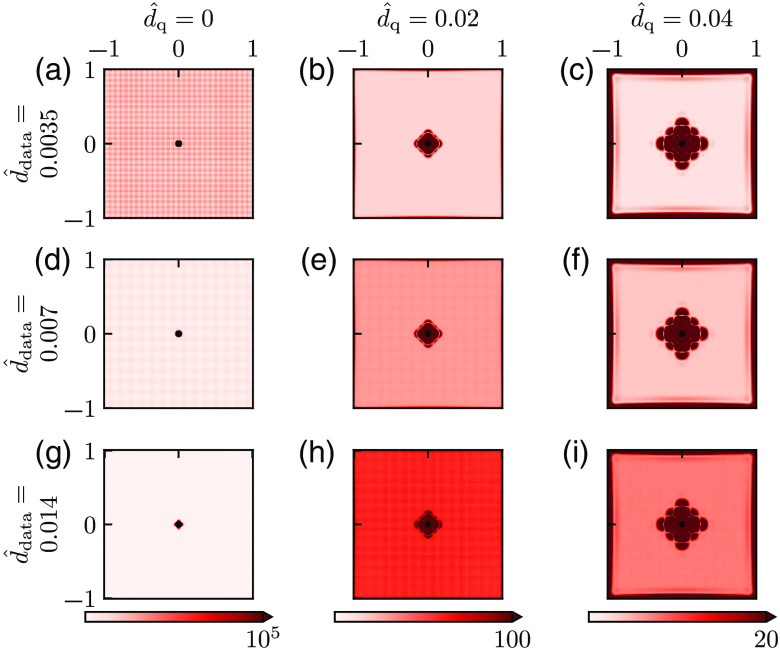
Root-mean-square error for different smoothing—fixed noise level. (a)–(i) RMSE computed from Eq. (21) for the bias and SDs shown in Figs. 4 and 5, respectively. Values are given as the percentage of the ground truth value $\hat{M}=1$.

Figure 6 shows the RMSE, computed from Eq. (22), for the example bias and SD shown in Figs. 4 and 5, respectively. For the smoothed cases (two right columns), we observe that the RMSE always increases with the d^data. Thus, with the noise level fixed, it is (unsurprisingly) always advantageous to have a dense measurement grid. For the noise level in this example, we see that the choice ${\hat{d}}_{q}=0.02$ (second column) gives a good estimate for d^data=0.0035, that is, low RMSE, for large parts of the map. For d^data=0.007 and especially d^data=0.014 the SD is not sufficiently reduced, and the RMSE is overall high. For the case with a larger smoothing (${\hat{d}}_{q}=0.04$, third column) the SD is much reduced for all values of d^data. However, the region with large bias around the vessel is increased, and the spatial region in which RMSE values are small is shrunken.

Note that the SD results in Fig. 5 and the RMSE results in Fig. 6 only pertain to the particular noise level used in the example, that is, ${\hat{\sigma }}_{P}=5\times 10^{-4}$. However, the SD is proportional to the noise level, so a doubling of ${\hat{\sigma }}_{P}$ would simply double the SD from what is shown in Fig. 5. RMSE results analogous to Fig. 6 for other noise levels can thus be obtained by appropriate scaling of SD in Eq. (22).

### 3.4 Estimation of CMRO_2_ for Other Example Situations

In the examples above, we have applied the diffusion-operator method to the situation with (i) a constant value of M and (ii) a single vessel providing the oxygen so that the ${\mathrm{pO}}_{2}$ map is described by the Krogh–Erlang formula in Eq. (16). For these examples, an alternative approach could be to estimate M by fitting the Krogh–Erlang formula directly to measured data.^4^ In other situations where, for example, M varies with position or several nearby vessels provide the oxygen so that the circular symmetry assumed in the Krogh–Erlang formula does not hold, this approach would not be applicable. In contrast, the current diffusion-operator method does not assume a constant M and can be applied to cases where multiple arterioles deliver oxygen.

#### 3.4.1 Spatially varying CMRO_2_

**Fig. 7:**
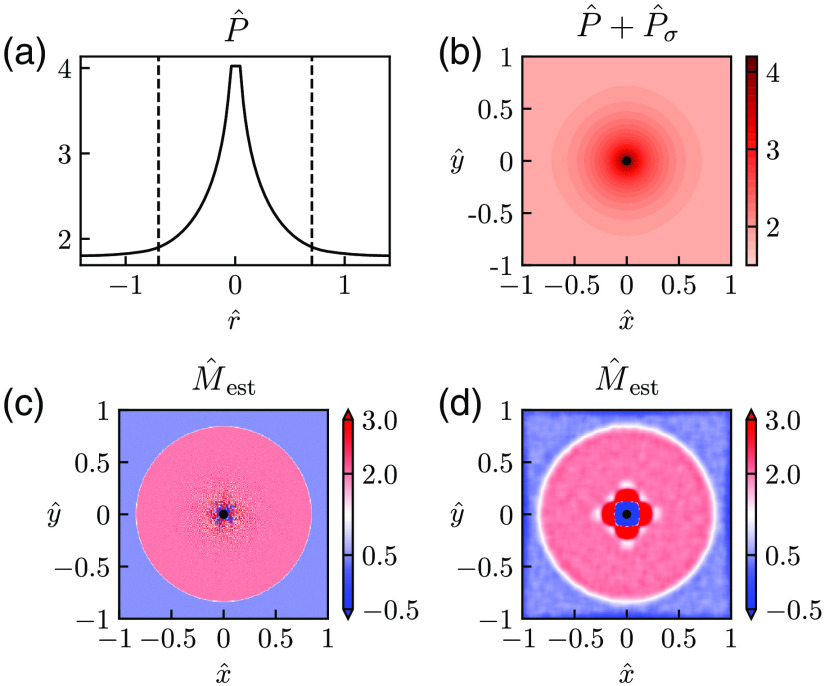
Estimation of spatially varying M. Diffusion-operator estimation of $\hat{M}$ for the case with a single oxygen-releasing vessel in the center with a larger $\hat{M}$ close to the vessel ($\hat{r}<0.7$ and $\hat{M}=2$) than far away ($\hat{r}>0.7$ and $\hat{M}=0.5$). The synthetic ${\mathrm{pO}}_{2}$ maps were calculated using the FEniCS numerical solver (see Sec. 2). (a) 1-D illustration of ${\mathrm{pO}}_{2}$ map for the case without noise (${\hat{\sigma }}_{P}=0$). The dotted lines mark the boundary between different levels of $\hat{M}$. (b) 2D illustration of the case with noise added (${\hat{\sigma }}_{P}=0.0005$). (c) Estimated $\hat{M}$ from the noise-less data without use of smoothing. (d) Estimated $\hat{M}$ from the data in (b) (where noise is present) with use of smoothing (${\hat{d}}_{q}=0.04$). Other parameter values: d^data=0.007, $P_{\mathrm{ves}}=80\;\mathrm{mm}\mathrm{Hg}$, $R_{\mathrm{ves}}=6\;\mu m$, $r^{*}=141\;\mu m$, and $M^{*}=10^{-3}$.

To illustrate the applicability of the Laplace estimator to the situation with varying M, we consider in Fig. 7 a hypothetical case where a single vessel provides the oxygen, but where the parameter M varies with distance from the vessel. Specifically, the value of M is assumed to be smaller far away from the vessel. This can be due to genuine differences in ${\mathrm{CMRO}}_{2}$. Alternatively, this can mimic the situation where a distant bed of capillaries acts as an oxygen source unaccounted for in the model and leading to an apparent decrease in ${\mathrm{CMRO}}_{2}$. Here the solution of the Poisson equation in Eq. (4) must be found numerically, and in Figs. 7(a) and (b), we illustrate the ${\mathrm{pO}}_{2}$ maps found using the FEniCS numerical solver (see Sec. 2). Figure 7(a) shows a 1-D representation of this ${\mathrm{pO}}_{2}$ profile in the radial direction for the case without any added noise. Figure 7(b) correspondingly shows a 2D colormap of the same synthetic data when noise has been added. The dotted lines in Fig. 7(a) mark the distance from the vessel ($|\hat{r}|=0.7$) where the value of $\hat{M}$ changes. With the characteristic length $r^{*}$ used throughout this paper, this corresponds to a physical distance of ∼100  μm, which is a typical size of the region around diving arterioles void of capillaries in the rat cortex.^4^ We see in Fig. 7(a) that beyond this distance, there is almost no decay in the ${\mathrm{pO}}_{2}$ compared to that within the capillary-free region.

When using the Laplace estimator on the noise-free data, we obtain excellent estimates of M, that is, ${\hat{M}}_{\mathrm{est}}\approx 2$ within the capillary-free region and ${\hat{M}}_{\mathrm{est}}\approx 0.5$ outside this region [Fig. 7(c)]. We only observe sizable errors in the immediate vicinity of the vessel, the errors stemming from the discreteness of the synthetic ${\mathrm{pO}}_{2}$ data used in the estimation (d^data=0.007). Further, when using the Laplace estimator on a smoothed version of the data in Fig. 7(b), we still obtain good estimates of $\hat{M}$ some distance away from the vessel. This is in agreement with the low values for the RMSE found for suitable smoothing of noisy data for the case with constant $\hat{M}$ in Fig. 6.

#### 3.4.2 Several vessels providing oxygen

**Fig. 8:**
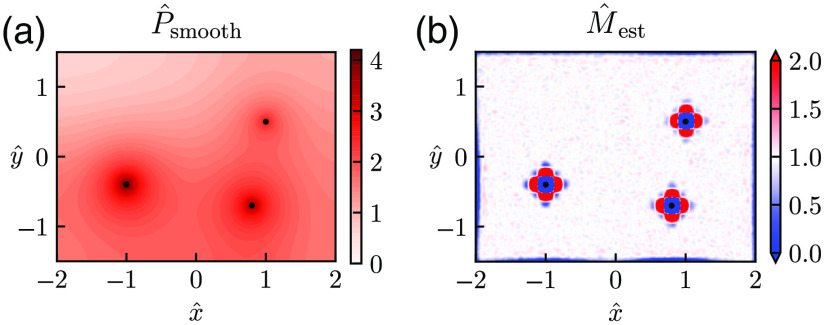
Estimation of M with several vessels providing oxygen. Example of diffusion-operator estimation for a situation where three vessels release oxygen into the tissue. The synthetic ${\mathrm{pO}}_{2}$ map was calculated using the FEniCS numerical solver (see Sec. 2). Here $P_{\mathrm{ves}}$ is set to 80, 70, and 50 mmHg for the vessel on the left, lower right, and upper right, respectively, whereas $R_{\mathrm{ves}}$ is set to 6  μm for all vessels. Noise is added to the synthetic data in (a) (${\hat{\sigma }}_{P}=0.0005$), and ${\hat{d}}_{q}=0.04$ is used in the smoothing to provide the estimates of $\hat{M}$ in (b). Other parameter values: d^data=0.007, $M=0.001\;\mathrm{mm}\mathrm{Hg}\;\mu m^{-2}$, $r^{*}=141\;\mu m$, and $M^{*}=M$.

An example of a situation where multiple nearby vessels serve as oxygen sources is shown in Fig. 8. Again, no analytical solution for the ${\mathrm{pO}}_{2}$ map is available, and the Poisson equation is instead computed by means of FEniCS. As observed in Fig. 8(a), the circular symmetry of the ${\mathrm{pO}}_{2}$ map seen in the earlier examples is broken around the vessels, but the Laplace estimator is still able to accurately estimate $\hat{M}$ except in locations close to the vessels [Fig. 8(b)].

### 3.5 Estimation of Spatially-Averaged M

So far, we have used the Laplace estimator to estimate spatial maps of M. The Laplace estimator can give accurate estimates as long as the noise level is not too large, but the estimates of M in the immediate vicinity of the oxygen-releasing blood vessels are typically inaccurate due to the bias introduced by the smoothing procedure.

In situations where the ${\mathrm{pO}}_{2}$ data are too noisy to give reliable spatially resolved maps of estimated M, one can still obtain estimates of spatially averaged values of M (as when estimating ${\mathrm{CMRO}}_{2}$ based on fitting the Krogh–Erlang model in Eq. (16) to experimental data^4^). The obvious procedure for estimating such average values $M_{\mathrm{est},\mathrm{av}}$ is to take the average over spatially resolved values of $M_{\mathrm{est}}$, that is

$$M_{\mathrm{est},\mathrm{av}}=\frac{1}{N}\overset{N}{\underset{i=1}{\sum }}M_{\mathrm{est}}(r_{i}).$$ (23)

The SD of $M_{\mathrm{est},\mathrm{av}}$ is then expected to be a factor $\sqrt{N}$ reduced compared to the SD for the spatially resolved estimates $M_{\mathrm{est}}(r)$.

**Fig. 9:**
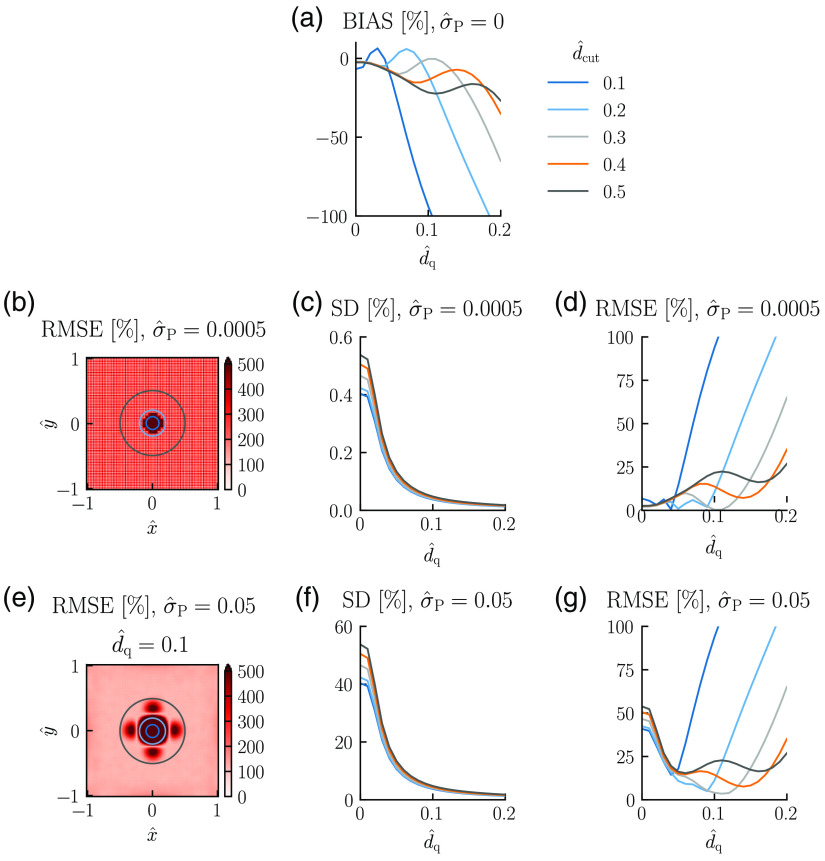
Estimation of spatially averaged M. Illustration of accuracy of the estimation of spatially averaged M for different values of the diameter d^cut of the circular disc removed from the average in Eq. (23). N=1000 has been used in the estimation of the SD [Eq. (20)]. Other parameter values: All panels: d^data=0.035, $P_{\mathrm{ves}}=80\;\mathrm{mmHg}$, $R_{\mathrm{ves}}=6\;\mu m$, $R_{t}=200\;\mu m$, $M=0.001\;\mathrm{mmHg}\;\mu m^{-2}$, $r^{*}=141\;\mu m$, and $M^{*}=M$. (a) ${\hat{\sigma }}_{P}=0$; (b)–(d) ${\hat{\sigma }}_{P}=5\times 10^{-4}$; and (e)–(g) σ^P=5×10−2, d^q=0.1. Note that for figure clarity, only the circles corresponding to d^cut=0.1, 0.2, and 0.5 are shown in (b) and (e).

The bias is not reduced by such an averaging procedure, however. To reduce the effects of smoothing-induced bias, one possible procedure is to take the average of M only for positions outside a circular region around the oxygen-delivering vessel. As illustrated in Fig. 9(a), this can reduce the bias in the $M_{\mathrm{est},\mathrm{av}}$ substantially. Larger values of the smoothing length ${\hat{d}}_{q}$ give larger regions of large bias around the vessel (Fig. 4). Thus larger areas around the vessel, parameterized by the diameter d^cut, should be removed from the averaging sum in Eq. (23) to keep the bias small. This removal of area from the averaging sum implies a smaller value for N in Eq. (23) and thus a larger value of SD of $M_{\mathrm{est},\mathrm{av}}$. Again, a compromise between the bias and the SD must be found to get the most accurate estimate.

This compromise is illustrated in Figs. 9(b)–9(g). Figure 9(b) shows the spatially resolved RMSE for a case with low noise corresponding to no smoothing applied (cf., left column of Fig. 6). Here the noise level is so low that even without smoothing, the SD of $M_{\mathrm{est},\mathrm{av}}$ becomes <1% for all averaging areas considered, that is, all choices of d^cut [cf., ${\hat{d}}_{q}=0$ in Fig. 9(c)]. With smoothing applied, the SD of $M_{\mathrm{est},\mathrm{av}}$ becomes even smaller, much <0.1% [Fig. 9(c)]. We also note that the SD is largest for the largest value of d^cut, reflecting that here the averaging area [and thus N in Eq. (23)] is the smallest. The corresponding RMSE is shown in Fig. 9(d). For this low-noise situation, there is nothing to gain by doing smoothing when estimating $M_{\mathrm{est},\mathrm{av}}$. The lowest RMSEs are obtained for ${\hat{d}}_{q}\approx 0$ since smoothing reduces the accuracy of the estimates due to the bias introduced [cf., Fig. 9(a)].

The situation with a much higher noise level (${\hat{\sigma }}_{P}$ a factor 100 larger, that is, ${\hat{\sigma }}_{P}=5\times 10^{-2}$) is shown in Figs. 9(e)–(g). The spatially resolved RMSE using a smoothing factor of ${\hat{d}}_{q}=0.1$ is seen to give large lobes with high RMSE values around the vessel [Fig. 9(e)]. Moreover, the typical RMSE value outside the lobe region is about 120%. The SD of $M_{\mathrm{est},\mathrm{av}}$ [Fig. 9(f)] is seen to be on the order of 50% for the case without smoothing (${\hat{d}}_{q}=0$), and a smaller RMSE can thus be obtained with smoothing applied [Fig. 9(g)]. The smallest RMSE, less than ∼10%, is obtained for ${\hat{d}}_{q}\approx 0.1$ and d^cut=0.3.

This high-noise example illustrates how accurate estimates of $M_{\mathrm{av}}$ can be obtained even when the spatially resolved estimates for M have a large uncertainty. With the parameter values used here, that is, $M^{*}=0.001\;\mathrm{mm}\mathrm{Hg}\;\mu m^{-2}$ and $r^{*}=141\;\mu m$, a ${\hat{\sigma }}_{P}$ of $5\times 10^{-2}$ corresponds to a physical noise level $\sigma_{P}$ of ≈1  mmHg. [Here we have used that $\sigma_{P}={\hat{\sigma }}_{P}M^{*}r^{*2}$, cf., Eq. (6).] For comparison, the corresponding ${\mathrm{pO}}_{2}$ at the vessel wall in this example would be $P_{\mathrm{ves}}=80\;\mathrm{mmHg}$.

## 4 Discussion

In this paper, we have introduced a new method, the diffusion-operator method, to provide spatially resolved maps of ${\mathrm{CMRO}}_{2}$ estimates based on two-photon measurements of ${\mathrm{pO}}_{2}$.^3^^,^^4^ The method has two key steps: (i) spatial smoothing of measured ${\mathrm{pO}}_{2}$ maps followed by (ii) application of double spatial derivatives in two spatial dimensions, that is, a Laplace operator. This method is an alternative to the Krogh–Erlang method where a spatially averaged value of ${\mathrm{CMRO}}_{2}$ is obtained around arterioles assuming circular symmetry.^4^

### 4.1 Choice of Inverse-Modeling Method

The present diffusion-operator method is an approach to the inverse diffusion problem in the context of ${\mathrm{CMRO}}_{2}$ estimation from high-resolution ${\mathrm{pO}}_{2}$ data obtained with two-photon microscopy. The two key elements of the method are (i) the Poisson equation in Eq. (4) describing how estimates of ${\mathrm{CMRO}}_{2}$, or more precisely the variable M(r) in principle can be found by applying the Laplace operator on measured ${\mathrm{pO}}_{2}$ maps $P_{\text{data}}(r)$ and (ii) the use of a smoothing routine on $P_{\text{data}}(r)$ to reduce effects of spatial noise before application of the Laplace operator. The development of the inverse-modeling method was mainly motivated by the need to have a method that is conceptually clear, easy to use, and based on publicly available software.

As the double spatial-derivative operation in the diffusion-operator approach is inherently sensitive to spatial noise, the choice of a suitable smoothing method is thus essential for obtaining accurate ${\mathrm{CMRO}}_{2}$ estimates. The ideal smoothing method should reduce the effects of this spatial noise without introducing large biases in the resulting estimates. We performed smoothing using the cubic smoothing spline function csaps from MATLAB’s Curve Fitting Toolbox. This method minimizes the square deviation between the estimated and measured data (so-called L2 norm) while penalizing large double-spatial derivatives in the smoothed ${\mathrm{pO}}_{2}$ maps [Eq. (10)]. However, other smoothing methods could be used, for example, with norms other than L2 or using different types of splines. Also since ${\mathrm{CMRO}}_{2}$, or more precisely the variable M in Eq. (4), is proportional to double spatial derivatives, the smoothing method inherent in csaps effectively penalizes large magnitudes of M and thus introduces an unwanted bias. An alternative approach could be to penalize instead changes in the spatial derivatives of M, that is, third spatial derivatives of the ${\mathrm{pO}}_{2}$. Finally, while csaps allows for different weighting of different locations within the map, the weighting functions are restricted to be spatially separable in the x and y directions. For the present application, this limitation is not optimal as it would be preferable to exclude only a small region in and around the vessel.

While the exploration of effects of different smoothing methods on estimation accuracy is beyond the current scope, an obvious next step would be to test the accuracy of the diffusion-operator method with other smoothing methods. In particular, it would be interesting to explore to what extent other methods could reduce the size and magnitude of the lobes of large bias seen around the vessel in Fig. 4. The present MATLAB scripts, which can be found online at https://github.com/CINPLA/CMRO2estimation, are designed to allow for an easy exchange of smoothing methods for such exploration.

### 4.2 Use of the Diffusion-Operator Method

The noise level and sampling distance in the experimental ${\mathrm{pO}}_{2}$ data reported in Ref. 4 were too large to allow for reliable estimation of spatially resolved maps of ${\mathrm{CMRO}}_{2}$ (results not shown). Further advancements in engineering of brighter and more sensitive optical ${\mathrm{pO}}_{2}$ probes and further development of optical instrumentation will improve the measurement accuracy^4^ and facilitate estimation of such maps. Additionally, other inverse-modeling methods may allow for more accurate spatially resolved ${\mathrm{CMRO}}_{2}$ estimation based on the same set of data.

Pooling of spatially resolved estimates [as described in Eq. (23)] will always improve the accuracy, but this will be at the expense of spatial resolution. This trade-off can be investigated within the present version or future variations of the diffusion-operator method using the scripts accompanying this paper. Estimation accuracy can be studied systematically with model-based ground truth data (either based on the Krogh–Erlang model or based on FEniCS simulations) using the same grid density and noise levels as those in the experimental setting.

### 4.3 Generalization of the Diffusion-Operator Method

Here the diffusion-operator method has been applied to estimation of ${\mathrm{CMRO}}_{2}$ for the case with 2D measurements of (assumed) steady state ${\mathrm{pO}}_{2}$ data. The diffusion-operator method straightforwardly generalizes to the 3D situation and also the nonstationary case where the ${\mathrm{pO}}_{2}$ varies with time. With time-resolved measurements of ${\mathrm{pO}}_{2}$ across a 3D volume of brain tissue, spatiotemporally resolved estimates of ${\mathrm{CMRO}}_{2}$ can be found by an analogous inverse-modeling problem based on Eq. (7). Also here, model-based validation of the estimation method can easily be pursued with synthetic data generated by finite-element modeling, for example, using FEniCS.
